# Evolution, Development, and the Incoherence of Sex: A Framework for Multiple Sex Concepts

**DOI:** 10.1093/icb/icag113

**Published:** 2026-07-10

**Authors:** Karen M Warkentin, Jay J Falk, A M Aramati Casper

**Affiliations:** Department of Biology, Boston University, 5 Cummington Mall, Boston, MA 02215, USA; Center for Animal Behavior, Smithsonian Tropical Research Institute, Apartado Postal 0843-03092, Panamá, República de Panamá; Department of Ecology and Evolutionary Biology, Princeton University, 11 Ivy Lane, Princeton, NJ 08544, USA; Center for Animal Behavior, Smithsonian Tropical Research Institute, Apartado Postal 0843-03092, Panamá, República de Panamá; Biology Department and Graduate Degree Program in Ecology, Campus Deliver 1878, Colorado State University, Fort Collins, CO 80523, USA

## Abstract

The word *sex* can refer to at least seven distinct, evolutionarily related biological phenomena (0–6 below). Bacteria and archaea use mechanisms for horizontal gene transfer (0) broadly and promiscuously, even without cell contact. Their endosymbiotic merger led to eukaryotes and a new form of gene exchange, using syngamy and meiosis (1) to mix and recombine similar genomes. This innovation altered the course of evolution. The requirement for chromosome homology in meiosis separated evolving lineages. Mating types evolved within them, differentiating roles of the cells pairing in syngamy, and gamete size dimorphisms (2) evolved many times. Organisms evolved diverse gamete-production strategies (3) and a plethora of traits associated with those strategies (4). They also evolved many ways to facilitate gamete encounters (5). Some of these were expressed in other contexts and gained new functions (6). These phenomena include cellular genetic processes (0 and 1), alternative states of cells and organisms (2–4), and things organisms do (5 and 6) that have diversified over billions of years. Sex is neither biologically simple nor conceptually singular, but the word is often used without qualifiers, assuming shared understanding that may not exist. We present a framework for multiple sex concepts that serve as anchor points to discuss the relationships among these phenomena and the diversity and complexity of each, including the biologically fuzzy edges generated by developmental variation and evolutionary change. We highlight several communication challenges that may limit biological understanding and/or facilitate the deployment of biology to justify social harms. For instance, *sex* is used for three alternative-state concepts (2–4 above) whose distinctions are sometimes collapsed, fostering overgeneralization based on the supposed simplicity of anisogamy, but even gamete sex is evolutionarily complex and subject to shifting definitional criteria. Attempts to narrowly bound “biological sex” minimize this complexity and what we can learn from it, while facilitating the misuse of biology in antidiversity social projects. Another challenge is that using accessible language that works for organisms like ourselves may misrepresent or obscure the biology of other life forms, but specialized language can create information silos; these limit the broad comparisons that are necessary both for perspective on our own biology and a more expansive understanding of life. The multiple sex concepts framework is a way to acknowledge the scope and discuss the complexity of sex in biology, offering a scaffold to facilitate broader thinking, better communication, and discovery.

Cover illustrations: (0) bacterial transformation and archaeal mating; (1) syngamy and meiosis; (2) anisogametes in green alga, fern, and mammal; (3) gamete production in sea anemone and fern gametophyte; (4) sex-differentiated antennae in moths, perfect and pistillate flowers in geraniums, and anglerfish female with small attached male; (5) treefrog amplexus, springtail transport of moss gametes, and coral broadcast spawning; and (6) orchid pollination by wasp pseudocopulation, female-on-male mounting in woodpeckers, and same-sex mating in dolphins (see Fig. 1 for framework).

**Fig. 1:**
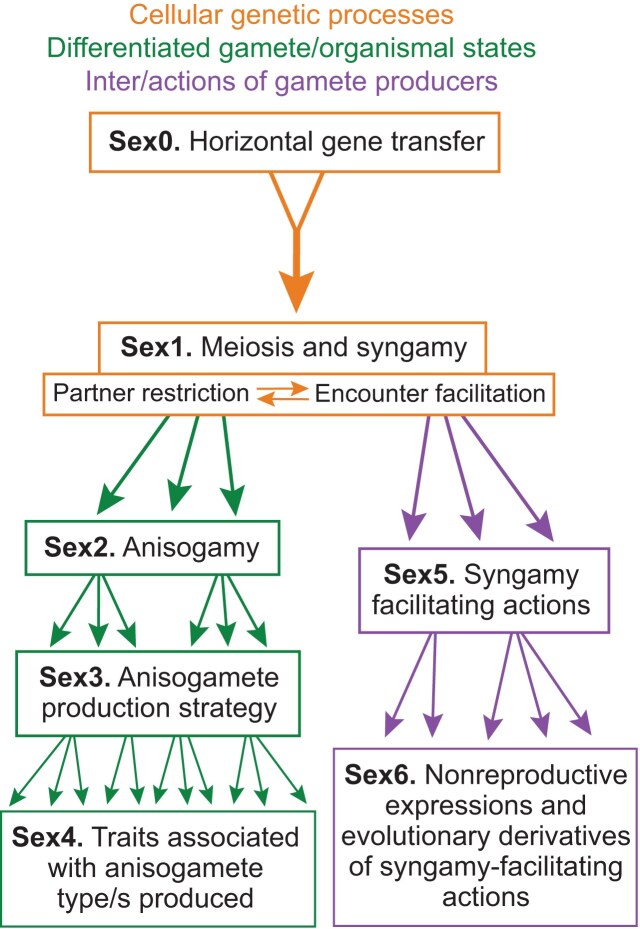
A framework for multiple sex concepts. Evolutionary relationships among biological phenomena sometimes called *sex* are indicated by arrows. Within cellular genetic processes, a converging arrow indicates multiple contributions of sex0 to the single origin of sex1. Multiple diverging arrows indicate multiple origins of sex2–6, with compounding layers of diversification within sexes (differentiated gametic and organismal states: sex2–4) and the actions and interactions of gamete producers (sex5 and 6).

## Introduction: The multiplicity, incoherence, and biological dynamics of sex

Biologically, the diversity and complexity of sex is amazing, and our understanding of it is growing rapidly. This dynamic richness is often absent from public discourse and undergraduate education, where oversimplified sexual biology is socially weaponized against gender and sexual minorities (Ritz and DuBois 2025) and alienating to students (Casper et al. 2022; Eddy et al. 2026). Overgeneralizations about sex persist, despite calls for more accurate representation of its diversity (Zemenick et al. 2022; Donovan et al. 2024) and broad efforts to compile and integrate information about sex (Jeffries et al. 2025; McDonough-Goldstein et al. 2026).

Effective communication is necessary to build shared knowledge and address social conflicts. However, there are a suite of biologically related but distinct phenomena for which we routinely use the same word, *sex*. These include cellular and genetic processes, alternative states of cells and of organisms, and actions and interactions of organisms. While the intended meaning of sex is sometimes clear from context, it is often unspecified or ambiguous, potentially causing misunderstandings. The author team discovered such misunderstandings among us multiple times, but only because we were collaborating on this manuscript. We now wonder how often they may occur without detection. Moreover, even when an intended central meaning of sex is clear, the extent of what is included may not be. In addition, multiple meanings of sex are routinely collapsed or equated, leading to overgeneralizations and further misunderstandings or inaccuracies.

Although the range of meanings of *sex* is much broader, recent debate focuses on alternative states, i.e., *sexes* (Eppley et al. 2026). Some biologists and philosophers of science have attempted to restrict the meaning of *sex/es* (Goymann et al. 2023; Griffiths and Spencer 2025). Other biologists highlight complexity and variation, suggesting an expansion (McLaughlin et al. 2023; Smiley et al. 2024), and historians have documented shifts and inconsistencies in usage of these terms (Velocci 2024). Given these inconsistencies, some have called for deconstructing, contextualizing, and even eliminating our use of *sex* (Richardson 2022; Massa et al. 2023; Watkins and DiMarco 2025). Calls to restrict and expand the meaning of *sex* both appeal to biological reality and social justice; however, despite partial overlap, each focuses on different aspects of biology (Goymann et al. 2023; McLaughlin et al. 2023). We need ways to discuss and think clearly about both—and about all the other biology encompassed by this one small word.

Here, we provide a framework of multiple sex concepts (Box 1, Fig. 1) and use them to discuss a set of biologically distinct processes and states called *sex*. These sex concepts are not alternative ways to define or operationalize a single biological phenomenon; they represent different phenomena. To more concisely and unambiguously discuss multiple sex concepts, we refer to them by numbers in this paper. Although we began using the numbers as a convenient shorthand, we found it helped us think broadly across life forms by disrupting our often animal-centric thinking cued by more standard phrasing, raising questions, and highlighting connections and discrepancies we had not previously noticed. In addition to addressing the biological scope and some of the complexities of sex, we use a series of examples to discuss ways that unacknowledged variation in language use can obscure or misrepresent biological variation, with implications for both the scientific and broader social understanding of sex.

We consider the sex concepts in our framework to be useful anchor points in thinking about the relationships among, and complexity within, these phenomena—not rigidly bounded definitions. While the central meaning of each concept is clear, particularly for familiar organisms like ourselves, its edges may not be. Rather than a “difficult boundary” (Fusco and Minelli 2019), we consider this a biologically fuzzy edge. Development generates variation around established traits, and evolution iteratively builds on that variation, making such fuzziness an inherent part of biological dynamics; indeed, fuzzy edges are often where such dynamics are most evident, making them rewarding research areas. However, specialists vary in how they operationalize concepts in these liminal zones, depending on their questions, and others may be misled by assuming congruent meaning.

Many aspects of biology are extremely divergent among lineages. Applying common, accessible language about sex that works well for familiar animals to organisms that differ, for instance in life cycle or body organization, may misrepresent or fail to encompass their biology. However, more accurate language is often taxon-specific, imposing a learning curve on nonspecialists. Restricting our thinking about sex to a limited set of familiar organisms constrains broader comparative analyses to assess diversity and/or convergence in solutions to shared problems. Moreover, particularly where human social biases shape our questions and knowledge about organisms more like ourselves, broader comparisons may improve our understanding of even familiar organisms.

Human historical and social biases have particularly impacted our understanding of sexes (Tang-Martínez 2016; Sandford 2023; Massa 2026), which has suffered from ambiguous usage, meaning slippage, and the collapse or assumed interchangeability of multiple sex concepts. We discuss the compounding layers of diversity in the evolution of such alternative states across organismal lineages and some areas of common oversimplification and overgeneralization. We also consider ways our communication challenges can limit biological understanding and suggest possible improvements.

Considering the interconnected and diversified scope of sex through the lens of multiple sex concepts focuses attention on the wonder of biology and the challenges inherent in human efforts to understand things so much larger than ourselves. For researchers, exploring biologically fuzzy edges and areas where our biases and communication challenges have limited understanding may be productive, revealing unsolved problems and raising new questions, offering useful test cases for general theory, shifting perspectives, and expanding our understanding of life. For teachers, this may be instructive to reveal science as a process, the limits and change of knowledge, and the complexity of life. For many, the ongoing discoveries of ways that life exceeds and complicates our prior knowledge may be inspiring.

Box 1. **Sex concepts representing seven distinct biological phenomena, grouped into three broad categories**

**I**. Cellular genetic mechanisms and processes

**Sex0**. Horizontal gene transfer: A diverse group of nonmeiotic mechanisms of genetic recombination including bacterial transformation, transduction and conjugation, archaean mating, and gene transfer in endosymbiosis.

**Sex1**. Meiosis and syngamy: A consolidated suite of eukaryotic cellular mechanisms for genetic recombination, DNA repair, and ploidy change; sexual vs. asexual.

**II**. Differentiated gametic and organismal states

**Sex2**. Anisogamy: Size-differentiated, self-incompatible macrogametes (large, female) and microgametes (small, male); sexed gametes vs. same-size isogametes.

**Sex3**. Anisogamete production strategy: Organismal capacity for/engagement in producing microgametes and/or macrogametes; female, male, hermaphrodite, or undefined/unsexed.

**Sex4**. Traits associated with anisogamete type/s produced: A species-specific multivariate hyperspace of genetic and phenotypic characteristics

**III**. Actions and interactions of gamete producers

**Sex5**. Syngamy-facilitating actions: The actions and interactions of gamete producers that bring compatible gametes into the proximity that enables syngamy

**Sex6**. Nonreproductive expressions and evolutionary derivatives of syngamy-facilitating actions: Includes byproduct expression of reproductive traits, exaptations involving new trait functions, and novelties derived from traits that originated for syngamy facilitation

## A framework of seven sex concepts

Here, we outline the structure of our sex concept framework (Box 1, Fig. 1) and relationships among the different concepts, before discussing each concept in more detail. The framework centers *meiosis and syngamy* (sex1) as the core eukaryotic cellular processes that enabled the evolution of many related phenomena (sex2–6). Meiosis recombines the homologous chromosomes of a diploid cell and assorts them to create novel haploid genomes and cells. Syngamy unites haploid genomes to make new diploids, usually through a sequential process of cell fusion (plasmogamy) and nuclear fusion (karyogamy). Sex1 anchors biological thought about sex, shapes eukaryotic life cycles, and is shared by descent across eukaryotes. However, it is not the first or most widespread form of genetic recombination. We include *horizontal gene transfer* (HGT) as sex0, in recognition of its importance for genetic variation, especially in prokaryotes (Van Etten and Bhattacharya 2020; Van Etten and Johnson 2026), and its role in the origin of sex1. Eukaryotes evolved via endosymbiosis between an archaean and a bacterium. Endosymbiotic gene transfer (one mechanism of HGT) contributed to conditions that favored sex1 in a stem eukaryote, and archaeal and/or bacterial genes involved in mechanisms of HGT provided key sex1 molecular machinery (Speijer et al. 2015; Moi et al. 2022); we illustrate this with a converging arrow in Fig. 1.

Meiotic recombination requires chromosomal homology, which breaks down as genomes diverge, imposing limits on genetic compatibility for reproduction. For syngamy to occur, cells must also be compatible, developmentally prepared to fuse (i.e., in a gametic state), and physically encounter each other. The interacting challenges of gamete encounter, gamete compatibility, and genome compatibility have shaped the evolution of gametes and the organisms that produce them in many ways.

Many lineages have evolved alternative self-incompatible intraspecific gamete classes that differ biochemically, morphologically, and/or behaviorally. The foundation for the evolution of differentiated sexes is *anisogamy* (sex2), in which compatible gametes differ in size. Anisogamy has evolved many times independently, among and within eukaryotic lineages. In anisogamous species, individuals may produce one or both types of gametes. Anisogamous lineages have evolved multiple ways to organize gamete production spatially and temporally, across singular (unitary) and modular body plans; we call these different *anisogamete-production strategies* sex3. Similarly, many lineages have evolved a variety of *additional differentiated traits that are associated with the anisogamete type/s an individual produces* (sex4). The types of traits involved and the ways they differ varies enormously, in lineage-specific ways. Sex2–4 all fit within the framework of sexes, with different alternatives states (female, male, and hermaphrodite variations). In Fig. 1, we use diverging arrows to illustrate multiple independent origins of the biological phenomena represented by a sex concept, and these layers of diversification compound.

The gamete encounters necessary for syngamy depend on the actions of both gametes themselves and the organisms that produce them. We call the *actions of gamete producers that facilitate syngamy* sex5. Different eukaryotic lineages have independently evolved many ways to bring gametes together, shaped by the constraints and opportunities generated by other aspects of their biology. Thus, this concept includes, but is not limited to, actions such as animal copulation that are commonly recognized as sex. For instance, the synchronized production of isogamous gametes by a unicellular alga in response to a local environmental cue and the visual display of flowers and release of odors that attract pollinators have likewise evolved under selection for bringing gametes together. Moreover, these syngamy-facilitation mechanisms, once evolved, can be expressed in other contexts and gain new functions. For instance, humans and other primates engage in nonreproductive sexual behavior for many reasons. *Nonreproductive expressions of, and traits evolved from, syngamy-facilitating actions* (sex6) could occur in any sexual lineage. Neither sex5 nor sex6 is restricted to anisogamous lineages. However, in anisogamous lineages sex2–4 certainly shape the evolution and expression of sex5 and 6, and vice-versa.

Overall, this sex concept framework articulates the multiple ways *sex* is used, describes each in an inclusive way that applies to organisms similar to and different from humans, distinguishes between usages that are sometimes confounded, and highlights the evolutionary relationships among these different biological phenomena. In the following sections, we discuss each sex concept and their relationships in more detail.

## Sex as cellular genetic mechanisms and processes

### Sex0. Horizontal Gene Transfer

HGT (sex0) involves the transfer of pieces of DNA between organisms without reproduction or complete genome exchange. Long before eukaryotes existed, prokaryotes were using multiple nonmeiotic mechanisms of HGT for genetic recombination; such gene exchange is common among prokaryotes and evolutionarily important in both prokaryotes and eukaryotes (Van Etten and Bhattacharya 2020; Mindell 2024; Van Etten and Johnson 2026). Bacteria transform themselves using environmental DNA from many sources, transmit plasmids to others through conjugation, and exchange DNA via viruses in transduction (Redfield 2001). These have all been called sex; transformation, specifically, is regulated by the recipient’s genome and the closest equivalent to eukaryotic sex (Ambur et al. 2016). Archaeans also mate, transferring chromosomes through cell bridges and by partial cell fusion and splitting (Gross and Bhattacharya 2010). Nonmeiotic genetic recombination enables DNA repair, shuffles alleles, and generates new gene combinations, conferring many of the benefits associated with meiotic sex (i.e., sex1). Moreover, key molecular mechanisms involved in prokaryotic sex0 are genetically homologous to those involved in eukaryotic sex1 (Speijer et al. 2015; Moi et al. 2022). Thus, sex0 is a clear evolutionary antecedent to the consolidated and central sex1 concept that follows and continues to play an important role in eukaryote evolution.

The origin of eukaryotes involved the endosymbiotic union of an Asgard archaean and an alphaproteobacterium; this created new evolutionary options and radically changed selection pressures, making many eukaryotic traits possible and necessary (Raval et al. 2022). Stem eukaryotes experienced a massive increase in genome size through endosymbiont gene transfer, HGT from other prokaryotes, gene duplication, and an invasion of self-splicing introns from the endosymbiont (Koonin 2006; Rogozin et al. 2012; Eme et al. 2017; Vosseberg et al. 2024). The proto-mitochondrial endosymbiont produced the energy to support larger genomes and cells, but also generated DNA-damaging reactive oxygen species inside those cells (Gross and Bhattacharya 2010; Hörandl and Speijer 2018; Speijer 2025). Prokaryotic sex0 is insufficient to prevent mutational decay in a large genome, particularly with more repeat sequences enabling misaligned recombination and consequent gene deletion. To maintain their expanded, repeat-rich genome, stem eukaryotes needed genome-wide homologous recombination of long stretches of DNA (Colnaghi et al. 2020; Colnaghi et al. 2022). This favored regulated fusion of genetically similar cells in syngamy. Moreover, genome-wide homologous pairing for DNA repair enabled the evolution of accurate homolog segregation in meiosis, unlike the random loss of chromosomes that occurs in prokaryotic ploidy cycles (Colnaghi et al. 2022; Gross and Bhattacharya 2010; Goodenough and Heitman 2014; Markov and Kaznacheev 2016; Fu et al. 2019). The merger of archaeal and bacterial genomes created the need for sex1. It also provided key molecular tools, some used in sex0, as precursors to the sex1 machinery for syngamy and meiosis (Schurko and Logsdon 2008; Speijer et al. 2015). For instance, the gamete-fusion protein HAP2/GCS1 is homologous to the archaeal fusexin protein Fsx1, which appears to mediate cell-fusion-dependent genetic exchange (Moi et al. 2022). Eukaryotic DMC1 mediates homologous DNA recombination in meiosis; its homologs archaeal RadA and bacterial RecA play a crucial role in prokaryotic DNA repair and recombination (Bernstein and Bernstein 2010; Matsuo et al. 2025).

Box 2. **Key terms in the biology of sex**

**I**. Sexual cellular/genetic processes

**Meiosis:** Sexual form of nuclear (and often cell) division; recombines and assorts homologous chromosomes of a diploid nucleus/cell to generate four haploid nuclei/cells with unique genomes

**Syngamy:** Usually, fusion of two haploid gametes to form a diploid zygote; more generally, merger of genetic material from two cells into a single genome

**Plasmogamy:** Fusion of cytoplasm, first step of syngamy

**Karyogamy:** Fusion of nuclei, final step of syngamy

**II**. Life cycles

**Haplontic:** Dominated by haploid phase; syngamy followed by meiosis, then many mitotic cell divisions

**Diplontic:** Dominated by diploid phase; meiosis followed by syngamy, then many mitotic cell divisions

**Haplodiplontic:** Major haploid and diploid phases; mitotic cell divisions after meiosis and after syngamy

**III**. Cells capable of syngamy

**Gamete:** Usually, a specialized reproductive cell that can fuse with another to form a zygote; more generally, a cell capable of giving and/or receiving a nuclear genome from another via permanent or temporary cell fusion

**Isogamy:** Usually, compatible gametes are the same size; more generally, compatibility in syngamy is not limited by gamete size class

**Anisogamy:** Compatible gametes are different sizes, same-size gametes cannot fuse in syngamy

**Oogamy:** Compatible gametes differ in size and mobility (small, mobile, or large, immobile); a specialized subset of anisogamy

**Macrogamete:** The larger of the two gamete types in anisogamy

**Microgamete:** The smaller of the two gamete types in anisogamy

**IV**. Sexual systems and individual sex conditions

**Gonochoristic:** Each individual can produce only macrogametes or only microgametes

**Hermaphroditic:** Each individual can produce both macrogametes and microgametes

**Mixed sexual system:** Includes gonochoristic and hermaphroditic individuals

**V**. Body plans

**Unitary:** Individuals are singular developmental/structural/functional units, with one sex condition

**Modular:** Organisms have multiple independent or semi-independent subunits, either physically attached or separated; sex condition may vary among subunits and individuality is complex

### Sex1. Meiosis and syngamy

Sex1 includes the fusion of haploid cells and genomes in syngamy to form diploids and the genetic repair, recombination, and ploidy reduction process of meiosis to form haploids (see Box 2 for key terms). From the first to last eukaryotic common ancestor, stem eukaryotes consolidated a suite of highly organized mechanisms for cell and nuclear fusion, DNA repair and recombination, and ploidy reduction that have been widely conserved ever since (Speijer et al. 2015). The origin of sex1 was a major innovation in the history of life, and this meaning of sex is conceptually central; its origin enabled the subsequent evolution of many other conditions and processes that we also call sex (i.e., sex2–6). While evolutionary biologists often treat sex1 as a single trait that can be maintained, lost, or expressed at some frequency (e.g., Otto 2009; Burke and Bonduriansky 2017), we consider it a highly consolidated suite of modular subunit traits (*sensu* West-Eberhard 2003) that are commonly inherited and expressed together, forming a higher-level unit of evolution, development, and function.

Although sex1 is consolidated, different elements can be expressed independently. Sexual eukaryotes have life cycles that include diploid and haploid phases. For instance, diatoms have a diplontic life cycle like animals, in which the diploid phase dominates and the only haploid cells are gametes, while *Chlamydomonas* algae have a haplontic life cycle, in which the haploid phase dominates and the only diploid stage is the zygote (Umen and Coelho 2019). In contrast, the sea lettuce *Ulva*, the kelps, and land plants all have haplodiplontic life cycles, with multicellular diploid sporophytes that make spores by meiosis and multicellular haploid gametophytes that make gametes by mitosis (Fusco and Minelli 2019; Umen and Coelho 2019). Thus, meiosis and syngamy occur in different generations of the haplodiplontic life cycle, with each life phase expressing a different subset of sex1 mechanisms. Nonetheless, sporophytes and meiotic spores are sometimes treated as “asexual” (Meissner 2021). A zoocentric, diplontic view of gametes—made by meiosis, capable of syngamy—as defining “sexual” fundamentally misunderstands haplodiplont sexuality and confuses their life cycles with metagenic ones, as in cnidarians, that include sexually and asexually reproducing generations of the same ploidy (Fusco and Minelli 2019).

Subsets of sex1 mechanisms have also been lost multiple times (Mirzaghaderi and Hörandl 2016). For instance parthenogenesis—a uniparental form of reproduction in which a gamete (often egg) develops without syngamy—has evolved many times. Sporadic parthenogenesis is documented in an ever-increasing list of reptiles and birds (Booth and Schuett 2016; Booth et al. 2023; Levine and Booth 2024). Cyclical parthenogenesis alternates with biparental sexual reproduction in many species of *Daphnia* and aphids, while other species only reproduce parthenogenetically (Decaestecker et al. 2009; Figueroa et al. 2018). The tropical night lizard *Lepidophyma smithii* produces mixed clutches of uniparental and biparental offspring (Kratochvíl et al. 2020) and at least 40 “all-female” reptile species only reproduce parthenogenetically, including the brahminy blind snake *Indotyphlops braminus* and many lizards, such as the desert grassland whiptail *Aspidoscelis uniparens* (Kearney et al. 2009). Parthenogenetic reproduction is often considered asexual and described as ameiotic, mitosis-like, or as females cloning themselves (Schön et al. 2009; Fujita et al. 2020; Jaron et al. 2021). However, many forms of parthenogenesis—in animals, perhaps all or most—involve a modified meiosis that maintains DNA repair functions, enables epigenetic resetting, and maintains or restores ploidy in a variety of ways (Gorelick and Carpinone 2009; Mirzaghaderi and Hörandl 2016; Cornaro et al. 2023; Blanc et al. 2025).

Several forms of reproduction involve gamete fusion with uniparental nuclear genome inheritance (Lehtonen et al. 2013), often involving a modified meiosis that generates diploid and/or anucleate gametes (Dedukh et al. 2022; Ross et al. 2022; Etoundi et al. 2024). In gynogenesis, females use sperm from related species to activate embryogenesis but discard the sperm genome. Many unisexual lineages of hybrid origin, such as the Amazon molly *Poecilia formosa*, use this strategy (Schlupp 2010; Ricemeyer et al. 2026). In androgenesis, sometimes called “male asexuality,” the offspring nuclear genome comes exclusively from the sperm. For instance, the endangered cyprus *Cupressus dupreziana* produces anucleate ova and diploid pollen; it can also use eggs of a related host species or serve as the ovule host for development of that species (Schwander and Oldroyd 2016). Hermaphroditic androgenetic lineages of invasive *Corbicula* clams produce diploid sperm that fuse with eggs of their own or related species, ejecting the egg’s genome (Etoundi et al. 2024). Like most hymenoptera, haploid males of the wasp *Messor ibericus* develop from unfertilized eggs. However, queens of this species also generate males of the allopatric *Messor structor*, which they need to sire their hybrid workers, by eliminating their own genome from eggs fertilized by *M. structor* sperm; this has been called xenogenesis (Juvé et al. 2025). *Bacillus* stick insects can reproduce through canonical sex1, but also parthenogenesis, gynogenesis, androgenesis, or even hybridogenesis, in which a lineage of hybrid origin selectively discards one parental genome during gamete formation, which is then replaced by mating each generation (Lavanchy et al. 2024). The water frogs *Pelophylax esculentus* are also hybridogenetic, and their populations differ in which parental genome is eliminated, based on which progenitor species is locally available to breed with (Ross et al. 2022). The unisexual *Ambystoma* salamanders, a hybrid polyploid complex with genomes from several sexual species, are considered the oldest asexual vertebrates. They can reproduce gynogenetically with males of five sexual species and also sometimes retain sperm genomes, either swapping out one of the maternal genomes (as in hybridogenesis) or increasing offspring ploidy (Bogart 2019).

**Fig. 2:**
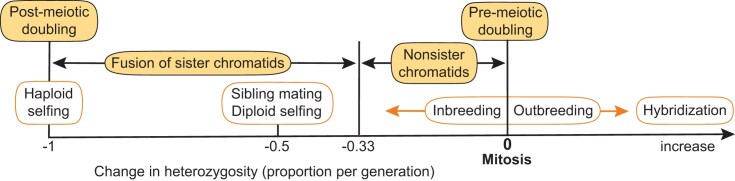
The complexity of sex1. The effect of “asexual” (top row, shaded) and “sexual” (bottom row, open) forms of reproduction on genetic variation can be similar or different; there is no simple binary. Change in heterozygosity across generations is shown for different mechanisms of meiotic parthenogenesis or androgenesis and patterns of mating, from a starting point (0) representing vegetative propagation (mitosis, in bold). Loss of heterozygosity (LOH) occurs through inbreeding and some forms of parthenogensis/androgenesis. LOH in terminal fusion, uniting sister chromatids, ranges from complete (−1) without recombination to as little as 1/3 with high recombination. In aborted meiosis I and central fusion, both uniting nonsister chromatids, LOH ranges from zero (i.e., genetically clonal reproduction) without recombination or with non-Mendelian segregation, to at most 1/3 with recombination and Mendelian segregation. Premeiotic doubling with sister chromosome pairing is genetically clonal (Blanc et al. 2025). Heterozygosity can increase through outbreeding; many parthenogens have high heterozygosity via hybrid origin.

Modified forms of meiosis may be considered forms of sex (Boyden 1950; Fusco and Minelli 2019) or quasi-sexual; alternatively, organisms may be considered asexual if they do not go through the full sex1 cycle with complete meiosis and syngamy (Bengtsson 2009). Moreover, eukaryotic microbes that lack male and female sexes also have variant forms of meiosis and development, some unique and others resembling the quasi-sexual strategies described above (Yadav et al. 2023). Importantly, the same genetic inheritance pattern can occur through different cellular mechanisms. Although some forms of parthenogenesis result in immediate or gradual loss of heterozygosity (Fig. 2), cytogenetic studies have revealed several modifications of meiosis that can maintain heterozygosity, resulting in genetic clonality (reviewed in Blanc et al. 2025). Reproduction involving syngamy, which is always considered sexual, can reduce genetic diversity through inbreeding, including sibling mating and self-fertilization by both diploid and haploid hermaphrodites. Outbreeding can increase heterozygosity. New parthenogenetic lineages often originate by hybridization, an extreme form of outbreeding, and are thus highly heterozygous. Overall, the genetic and evolutionary consequences of different forms of reproduction vary along a spectrum (Fig. 2) that is obscured by a binary division into sexual vs. asexual.

Many organisms have two or more reproductive options, including but not limited to full sex1. For instance, most protists usually reproduce mitotically and only occasionally use sex, while plants often reproduce both vegetatively and sexually. Such species are often grouped with obligately biparental species as sexual, rather than distinguished as reproductively bimodal. However, the capacity for various forms of uniparental reproduction is both demographically and evolutionarily important, and reproductive bi- or multimodality is prevalent across eukaryotes (Fusco and Minelli 2019; Rizos et al. 2024). Binary thinking about sexual vs. asexual reproduction likely fueled the intensity of interest in the “paradox of sex,” which only recently shifted to focus on obligate sexual reproduction (Otto 2009; Burke and Bonduriansky 2019; Wilner et al. 2025). It also likely slowed our recognition of facultative parthenogenesis in vertebrates (Levine and Booth 2024). Moreover, treating sex1 as a form of reproduction—which it is in multicellular eukaryotes—ignores eukaryotic lineages such as ciliates, in which sex1 creates new genetic combinations but not more individuals (Jiang et al. 2019). Indeed, sex1 is often associated with temporary cessation of reproduction in protists (Rizos et al. 2024; Constable and Liu 2026).

Although the central meaning of sex1 is anchored in canonical meiosis and syngamy, the edges are fuzzy. There is no consensus on which elements of meiosis and syngamy are necessary and sufficient for a process to be considered sexual, and different subsets are relevant for different questions. Considering sex1 as a consolidated modular trait, constructed from the coevolution and coexpression of a suite of semi-independent subunit traits across life cycles, might be useful for studying both the origin of sex1 and variation in its maintenance and expression. This theoretical evolutionary framing (West-Eberhard 2003) would be consistent with the ongoing dissection of the molecular evolution of cytogenetic mechanisms of meiosis and syngamy as well as biological thought about other complex traits. The fact that sex1 is shared by descent from a eukaryotic common ancestor makes it the most consistent, cohesive, and general meaning of sex. Variation in where biologists draw boundaries around it does not conflict with the biological unity of the central concept.

Although the diverse mechanisms of sex0 enable genetic exchange among extremely divergent lineages, meiosis requires chromosome homology. Thus, sex1 requires mechanisms to restrict syngamy to genetically similar cells, isolating evolving lineages. Although sex0 transformation using environmental DNA can occur without cell contact, combining entire genomes in sex1 requires temporary or permanent cell fusion. Thus, sex1 also requires ways to bring compatible cells together in space and time for syngamy. There are many interacting mechanisms that restrict gamete pairing and facilitate gamete encounter, including shared responses to external sex cues, pheromone–receptor systems, and compatible cell-surface proteins (Rizos et al. 2024). The shared and compatible traits that enable syngamy also block incompatible cells from fusing, contributing to species barriers. However, the eukaryotic common ancestor probably lacked intraspecific restrictions on syngamy, so partners could be genetically and morphologically identical (Goodenough and Heitman 2014; Heitman 2015).

## Differentiated gametic and organismal states

Many eukaryotes—including protists, algae, fungi, and more—still have size-matched (isogamous) gametes, and some have universal gamete compatibility (Heitman 2015; Rizos et al. 2024). However, eukaryotic lineages have evolved many intraspecific mechanisms to restrict syngamy partners, based on biochemical, morphological, and behavioral compatibilities of gametes and gamete-producers. In isogamous species, biochemical mechanisms regulating cell–cell attraction and fusion often create distinct self-incompatible mating types (Goodenough and Heitman 2014). Some species have just a few self-incompatible types, often two, but others have many (Heitman 2015; Constable and Kokko 2018; Wallen and Perlin 2018). For instance, most ascomycete fungi have two mating types, although self-compatibility has evolved many times (Billiard et al. 2011). In contrast, many basidiomycete fungi have large numbers of mating types, regulated by two loci that each have many alleles; for instance, *Shizophyllum commune* has at least 28,000 mating types, each compatible with most of the others (Wallen and Perlin 2018). The ciliate genus *Tetrahymena* includes asexual species that do not engage in sex1, a “unisexual” species with obligate selfing, and species with 3–9 mating types (Yan et al. 2021). Isogamous mating types are sometimes called sexes, particularly in binary systems (Leonard 2010), but usually not. Moreover, some have argued that differential transmission of organelle genomes (e.g., in mitochondria) should be the defining feature of sexes; this also evolved in isogamous species (Hurst and Hamilton 1992; Lane and Pomiankowski 2026). Here, we will follow the common convention of using *sexes* only for size-differentiated binary mating types.

Three sex concepts (sex2–4), all sometimes called “biological sex,” relate to the subset of syngamy-restriction systems called anisogamy, in which compatible gametes differ in size. These three concepts of sex are entwined and codependent; their meanings are neither dissociable nor equivalent. They are often assumed to be biologically linked in ways that foster interchangeability, and intended meaning is rarely specified. However, compaction and slippage of meaning across these concepts can lead to inadvertent misunderstanding and is also being weaponized to justify harms to sex and gender minorities (Ritz and DuBois 2025). Sex2–4 all include alternative states called female and male. Sex3 and 4 include additional states called by a variety of specialized terms. When individuals produce only one type of sexed gamete, we will refer to them collectively as gonochorists. When individuals produce both types of sexed gamete, concurrently or sequentially, we will refer to them as hermaphrodites, using adjectives to clarify specifics. After discussing each state-based sex concept separately we will consider them together.

### Sex2. Anisogamous gamete size classes

**Fig. 3:**
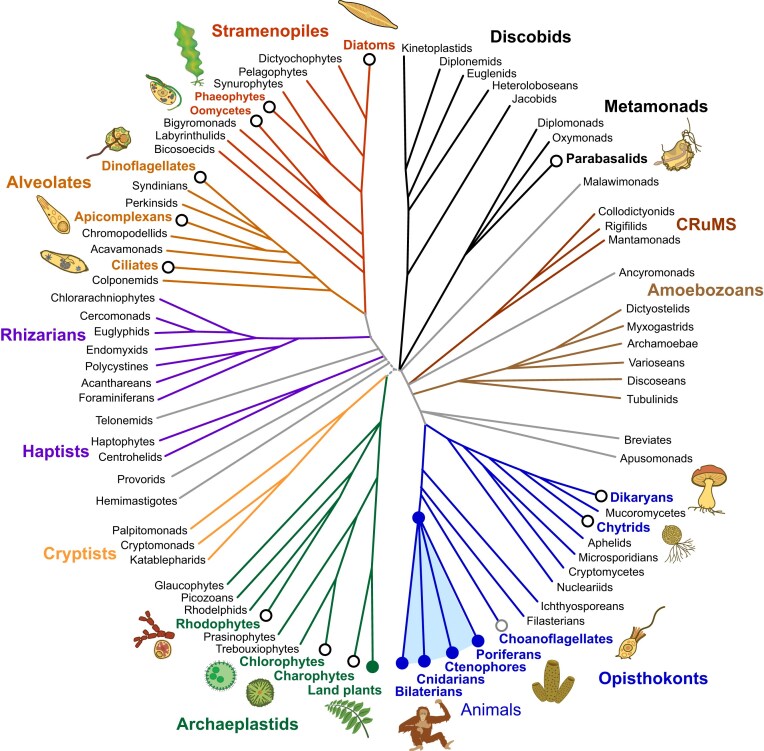
The multiple origins of sex2 across eukaryotes. Most eukaryotic lineages are unicellular and isogamous. Filled circles indicate ancestrally anisogamous clades representing two origins with pervasive anisogamy (animals, grouped by shaded triangle, and land plants). Black open circles indicate clades with both isogamous and anisogamous species; some include multiple gains and/or losses of anisogamy. Grey open circle indicates possible anisogamy and isogamy in choanoflagellates. See text for citations and note that not all origins of anisogamy are included. Phylogeny and thumbnails are open access from Keeling and Eglit 2023, except chytrid, sponge and *Volvox* by K.M.W.

Binary, size-differentiated, self-incompatible gametes (anisogamy or sex2) have evolved independently many times. Animals and land plants are each ancestrally and broadly anisogamous, but these two origins are not representative of eukaryotic sexual diversity. Isogamous and anisogamous lineages are interspersed throughout the eukaryote phylogeny (Fig. 3), and many closely related taxa differ (Keeling and Burki 2019; Rizos et al. 2024). For instance, dikaryan and chytrid fungi, diatoms, dinoflagellates, ciliates, apicomplexans, parabasalids, and many clades of algae all include isogamous and anisogamous taxa (James et al. 2006; Billiard et al. 2011; Togashi et al. 2012; Hanschen et al. 2018; Fusco and Minelli 2019; Umen and Coelho 2019; Choi et al. 2024; Berger et al. 2026). These represent an unknown but large number of origins and secondary losses of anisogamy, offering excellent opportunities for comparative research. In choanoflagellates, the closest protist relatives of animals, one species engages in anisogamous syngamy under nutrient limitation (Levin and King 2013) and what appears to be isogamous syngamy in response to a chemical cue from bacteria (Woznica et al. 2017; Woznica and King 2018).

Across origins, we define the larger gamete as female and the smaller as male, although their absolute sizes and how much they differ vary substantially across taxa. Anisogamy may usually evolve by adding size dimorphism to pre-existing binary mating types (Lehtonen and Kokko 2011; Togashi and Cox 2011), but they need not be linked. For instance, several *Chlamydomonas* species with binary mating types have two modes of gametogenesis: Gamete-producing cells can differentiate into one large gamete or make multiple small gametes through 2–3 mitoses (Wiese et al. 1979). Which gamete size they produce is a plastic trait, independent of mating type, so syngamy can occur between same- or different-sized cells and the species are considered isogamous. Thus, a binary gamete size difference does not inherently generate self-incompatibility. Moreover, anisogamy can coexist with additional self-incompatibility systems, creating multiple axes of syngamy restriction (Billiard et al. 2026), as in ascomycete fungi and many hermaphroditic plants (Ni et al. 2011; Bedinger et al. 2017; Endress 2020; Broz and Bedinger 2021).

Anisogamous organisms have evolved many lineage-specific, size-linked gamete traits, including differences in morphology, mobility, signaling, sensing, and organelle inheritance (Leonard 2010). However, such traits are neither necessary nor sufficient to identify anisogamy. For instance, asymmetric signaling interactions are common in isogamous mating types (Hadjivasiliou and Pomiankowski 2016). Organelle inheritance can be uniparental in isogamous species and, although commonly maternal, it can also be biparental or paternal in anisogamous species (Camus et al. 2022; Lane and Pomiankowski 2026). For instance, many bivalves have doubly uniparental mitochondrial inheritance; eggs transmit maternal mitochondria and sperm transmit paternal mitochondria, which persist in the male gonad (Soroka 2020). In bananas and cucumbers, mitochondrial inheritance is paternal; this is also the case for some strains of an anisogamous brown alga, *Ectocarpus*, but in other strains mitochondria are inherited randomly from either parent (Mignerot et al. 2019). The subset of anisogamy in which macrogametes are immobile and microgametes are mobile is called oogamy and sometimes distinguished from anisogamy *sensu stricto*, in which both gamete types are mobile (Fusco and Minelli 2019). However, among ancestrally oogamous lineages, seed plants have lost sperm mobility (Becker et al. 2025) and some kelp have evolved apparently functional egg flagella (Choi et al. 2024).

Gamete interactions, including competition for and difficulty encountering syngamy partners, can create functional trade-offs that disfavor intermediate-sized gametes; this is thought to drive the evolution of anisogamy and further elaborations in gamete dimorphism (Parker et al. 1972; Togashi and Cox 2011; Lehtonen et al. 2016). Although earlier work argued that initial divergence would lead inevitably to increasing dimorphism and the accumulation of specialized sex-specific traits, it is empirically clear that gamete divergence can also be reversed. For instance, some green algae have lost oogamy, returning to anisogamy *sensu stricto* (Hanschen et al. 2018), and brown algae have reverted from oogamy to isogamy multiple times (Choi et al. 2024). Thus, more recent work considers additional contextual factors in gamete evolution. For instance, gamete parthenogenesis is widespread in both green and brown algae, occurring in macro- and isogametes and, surprisingly, also in microgametes of some anisogamous and oogamous species (Heesch et al. 2021; Lehtonen et al. 2021). Moreover, in some ascomycete fungi, including the bread mold *Neurospora crassa*, the same small haploid cells can either function as male gametes or develop into haploid hyphae (i.e., function as spermatia or conidia, Ni et al. 2011; Kirschner 2019). Including gamete parthenogenesis in models expands the range of evolutionary outcomes (Constable and Kokko 2021; Lehtonen et al. 2021), predicting the large isogametes that occur in algae but had been hard to explain (Togashi et al. 2024). Moreover, modeling a range of gamete densities and empirically supported relationships of competitive traits with gamete size suggests anisogamy can coevolve with greater investment into competition by either microgametes or macrogametes, under conditions common in marine broadcast spawners (Levitan 1993; Seed and Tomkins 2018; Siljestam and Martinossi-Allibert 2024). Thus, the emerging picture—empirically and theoretically—is of greater variation in gamete traits and their evolution than expected under classic theory.

The meaning of sex/es is contested, but most arguments focus on gametic (sex2–3) vs. organismal (sex4) meanings (Eppley et al. 2026). However, not even gamete sex definitions are stable. If size-dimorphism of self-incompatible gametes is the foundation defining males and females (Parker et al. 1972; Lehtonen et al. 2016; Goymann et al. 2023), then any loss of defining features should equate to the loss of sexes. Identification and analysis of trait gains and losses is standard practice in evolutionary biology. For instance, powered flight evolved independently in bats, birds, and insects, was subsequently lost many times in birds and insects, and has—at least in insects—occasionally reappeared (Whiting et al. 2003; Maderspacher 2022). However, biologists do not consistently apply this standard practice to sexes. When anisogamous ancestors give rise to isogamous descendants, their equal-sized gametes may retain differentiated, formerly size-associated traits. Switching from a trait-based to an inferred lineage-based definition of sexes allows such gametes to be identified as “male” or “female” based on traits such as activity level or persistence in signaling (Heesch et al. 2021; Choi et al. 2024). Similarly, many obligate parthenogens descended from the females of biparental species are still called “female,” despite their lack of male syngamy partners and self-compatible meiotic products (Boyden 1950; Schön et al. 2009; Gorelick et al. 2017). Such shifts in meaning prioritize historical continuity over a consistent definition of sexes; they foster an illusion of sex2 stability and, when phylogenies are uncertain, might falsely identify “males” and “females” in some cases of ancestral isogamy.

Ideally, gamete sex (or lack thereof) should be identifiable based on consistent, measurable gamete traits. It could be useful to consistently distinguish size-based gamete dimorphism as anisogamy, with micro- and macrogametes, avoiding the imprecise language of sexes, male, and female. When a focal species with same-size gametes is nested within a clade with size-differentiated gametes, terms such as *ancestrally anisogamous* or *derived isogamy* convey historical information without introducing confusion about current traits. To discuss multiple differentiated gamete traits sometimes used to identify “sex,” one approach could be to subdivide sex2 into a strictly size-based concept (sex2A) and use sex2B, 2C, etc. to indicate other relevant and measurable axes of variation in gamete traits such as mobility, mitochondrial inheritance, chloroplast inheritance, etc. Recognizing these as distinct, noninterchangeable axes of gametic variation would facilitate analyses of the strength and patterns of correlations among them. Importantly, all of these “gamete sex” traits show changes after their origins in some lineages; none is consistently stable. Moreover, their canonically sexed directionality sometimes conflicts—as in bananas, with immobile sperm that transmit mitochondria and eggs that transmit chloroplasts (Camus et al. 2022).

However, the incoherent use of sexed language in biology is larger than the problems of size vs. organelle inheritance, current size vs. lineage, or lineage uncertainty. This language is also applied to intracellular components in isogamous species, such as basidiomycete fungi and ciliates that bidirectionally exchange haploid nuclei through conjugation, treating migratory nuclei as “male” and stationary ones as “female” (Chau and Ng 1988; Vreeburg et al. 2016). This may be based simply on historical precedent and old ideas of an active male essence (Sandford 2023). “Maternal” and “paternal” has even been applied to organelle inheritance in isogamous protists, such as *Chlamydomonas*, in which chloroplasts and mitochondria are transmitted by different mating types (Aoyama et al. 2006). Unlike the biologically fuzzy edges of sex1, inconsistent usage around sex2 represents a biased form of meaning slippage. Defining sexes by a size difference in syngamy partners enables biologists to identify them in any lineage where such a difference exists, regardless of other gamete traits; using additional gamete traits to also define sexes expands the set of included species and can obscure reversions to isogamy. This makes sexes appear more stable and more widespread, but less coherent, than adherence to the foundational definition would support.

Sexes evolved independently in lineages that already differed in many traits, and thus in their evolutionary and developmental potential. The selection pressures that favored anisogamy may also have varied, shaping additional derived traits of sexed gametes (Lehtonen and Kokko 2011; Siljestam and Martinossi-Allibert 2024). Across origins, sex2 has persisted for different lengths of time, through different conditions, with different opportunities for entrenchment, diversification and loss, and homologous sexed gametes have diversified enormously in some cases (Fitzpatrick et al. 2022). Gorelick et al. (2017) found no universal features distinguishing male from female gametes across eukaryotes or even within animals, plants, or brown algae. For instance, giant sperm have evolved in many animal phyla, and some seed plants have very small eggs (Gorelick et al. 2017). Considering the analogous nature of sexes across origins, and flexibility with which some biologists apply sex-defining characteristics, this incoherence is not surprising. However, it conflicts with the idea that anisogamy is a truly general phenomenon, with consistent evolutionary consequences, and cautions against overgeneralizations about sex2, or males and females, based on studies of a limited subset of organisms. The multiple origins of sex2 should enable robust comparative research to determine what (if any) generalities about gamete sexes truly exist; however, the success of comparative analysis depends on clear, consistently applied trait definitions.

### Sex3. Anisogamete production strategy

**Fig. 4:**
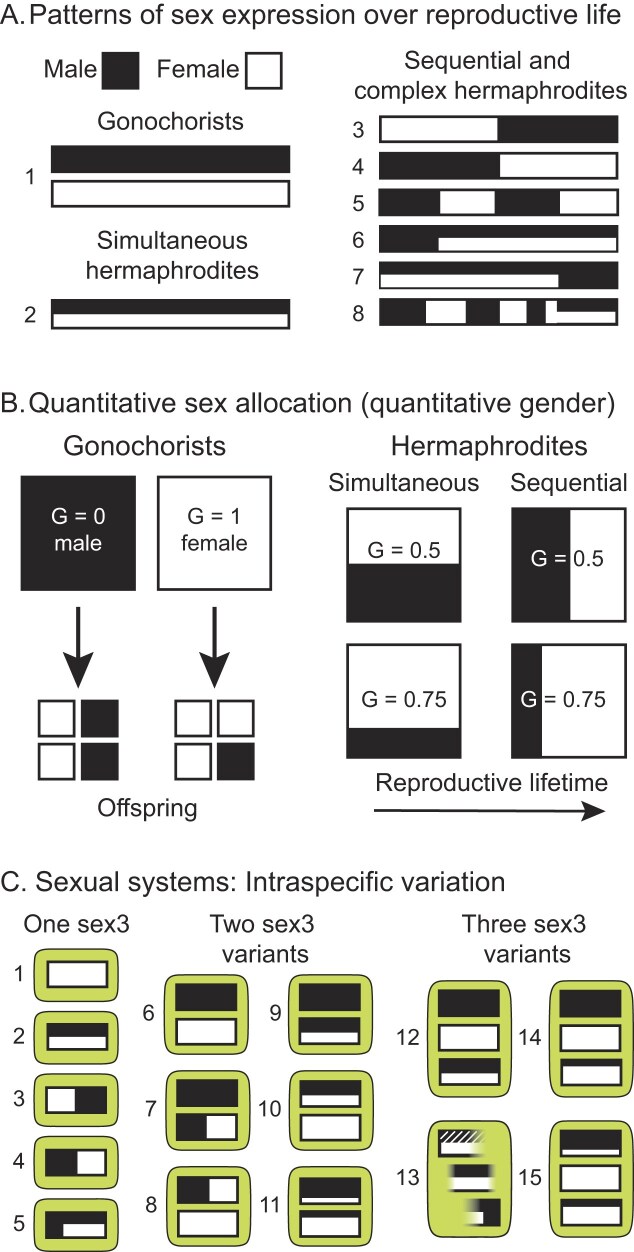
The complexity of sex3. The organization of anisogamete production in bodies. (A) Patterns of microgamete (black) and/or macrogamete (white) production across reproductive lifetime: (1) gonochorists, (2) simultaneous hermaphrodites, (3) protogyny, (4) protandry, (5) bidirectional sex change, and (6–8) more complex patterns of hermaphroditism (see text). (B) Quantitative sex allocation in gonochorists and hermaphrodites. Gonochorists produce only microgametes or macrogametes and invest resources equally (left) or differentially (right) into offspring sexes. Hermaphrodites invest equally (top) or differentially (bottom) in microgamete and macrogamete production, simultaneously or sequentially. G is phenotypic quantitative gender. (C) Sexual system diversity, showing examples of variation in the number and kinds of sex3 within species (shaded enclosures). One sex3 kind: (1) obligate parthenogens, (2) simultaneous hermaphrodites, (3) protogyny, (4) protandry, (5) protandric simultaneous hermaphroditism. Two sex3 kinds: (6) gonochorism, (7) protandry with permanent males, (8) protandry with primary females, (9) androdioecy, (10) gynodioecy, and (11) simultaneous hermaphrodites with sex allocation variants. Three sex3 kinds: (12) trioecy and (13–15) more complex sexual systems. (13, top) represents functionally female phenotypic hermaphrodites, with unused male function (hatched); blurred boundaries represent unknown past or future states. See text for examples of different kinds of hermaphrodites and sexual systems.

While sex2 pertains to characteristics of syngamy-capable cells, the organisms that produce anisogamous gametes vary in which type/s they produce, sometimes called sex condition or gametic sex; we call this sex3. In a broad sense, considering entire lifespans and underlying potential, sex3 takes four forms: Individuals can produce macrogametes (female), microgametes (male), both gamete types (hermaphrodites), or neither (unsexed). Hermaphrodites can produce both micro- and macrogametes simultaneously, sequentially, or in some more complex ontogeny (Fig. 4; Leonard 2018). Isogametic species that lack sex2 have no defined sex3, and neither do permanently sterile castes in eusocial species such as termites and ants. A variant of the sex3 concept focuses only on current gamete production, so most individuals lack a sex3 for part of their life; these include embryos and larvae, sequential hermaphrodites in transition, post-oopausal individuals, neutered pets, and more (Urfer and Kaeberlein 2019; Griffiths 2021; Winkler and Goncalves 2023). In this model, strictly sequential hermaphrodites are considered male and female at different times. Both lifetime potential and current gamete production are biologically important in different contexts and for different questions.

For gonochorists, sex3 seems simple, and strictly simultaneous hermaphrodites may be only slightly more complex (Fig. 4A1 and 2). However, these are just the extremes of a rich diversity of sex3 expression patterns (Scharer 2017; Leonard 2018). Sequential hermaphrodites can change sex from female to male (protogyny, Fig. 4A3) as in wrasses, from male to female (protandry, Fig. 4A4) as in clownfish, or bidirectionally (Fig. 4A5) as in coral gobies (Kuwamura et al. 2020). The production of each gamete type can be finely timed to increase reproduction, in response to environmental or social context (Picchi and Lorenzi 2018) or organismal state. “Thus, *Ostrea* oysters are only male while pregnant” (Leonard 2013); they switch from egg production to sperm production while brooding embryos, then return to egg production once larval exit makes space available in their body (Fig. 4A5). In some species, individuals can spend part of their life as one sex and part as simultaneous hermaphrodites (Fig. 4A6 and 7). Lysmatid shrimp mature as males then gain female function, becoming simultaneous hermaphrodites (Baeza 2018), while seabass *Serranus baldwinii* mature as hermaphrodites but large individuals can become male later in life (Petersen 2006). Repeated bidirectional sex change can also grade into simultaneous hermaphrodism (Fig. 4A8). In polychaete *Ophryotrocha puerilis* pairs, larger females lay more eggs but males grow faster; when a male outgrows its mate both switch sex and if pairs are together long enough they begin to function as simultaneous hermaphrodites that alternate roles at each mating (Berglund 1986). Sex3 expression has a history of many transitions among gonochorism and various forms of hermaphrodism (Kerr et al. 2011; McDaniel et al. 2013; Sasson and Ryan 2017; Leonard 2018; Wang et al. 2021; Pla et al. 2022). Such changes necessarily begin within species, with individual developmental variation (West-Eberhard 2003), thus not all species or individuals fit clearly into our categories.

Rather than categorically change sex, hermaphrodites often quantitatively shift their investment into micro- and macrogamete production. The concept of quantitative gender (Fig. 4B) (Lloyd 1980; Pannell 2023) was developed to describe this continuous variation in sex allocation (Charnov 1982; West 2009) within an individual more specifically than categorization as male, female, or hermaphrodite allows. This usage of the word *gender* is distinct from its human, social meaning; for an extensive discussion of the history and usages of *gender* see McDonough-Goldstein et al. (2026). Quantitative gender can be used to describe sexual phenotypes or reproductive function proportionally, from 0 to 1. Botanists generally use *phenotypic gender* to measure proportional investment into macrogamete production (1 = female). Zoologists use phenotypic or *gonadal gender* similarly or as proportional investment into microgametes (1 = male) (McCartney 1997; Leonard 2010). However, gamete production is distinct from gamete use; for instance, individuals can be phenotypically hermaphroditic but functionally male or female, at a particular time or throughout their life (Mayer and Charlesworth 1991; Pannell 2002; Picchi and Lorenzi 2018). Again, usage varies. Plant *functional gender* represents the proportion of offspring produced via macrogametes, which depends on both phenotypic gender and the local mating environment (Lloyd 1980; Pannell 2023). For instance, a plant with a phenotypic gender of 0.5 in a highly female-biased population would have a functional gender close to 0. In zoology, functional or *behavioral gender* can refer to mating performance, not offspring; an animal with gamete-producing testes and ovaries may mate in both sex roles or only in one (St. Mary 2000). Sex allocation theory also pertains to gonochorists, which produce one gamete type but quantitatively invest into male and female offspring (West 2009). When measured in sporophytes, plant quantitative gender is also a cross-generational property, reflecting investment in (or offspring via) macrogametophytes which make macrogametes. Thus, beyond simple categories, sex3 can be a quantitative trait that varies contextually within and among individuals.

Moreover, individual expressions of sex3 coexist within populations and species in combinations called sexual systems (Leonard 2018) (Fig. 4C). Species may have only a single sex3 type, as in all-female parthenogens and various forms of hermaphroditism (Fig. 4C1–5). Males and females coexist in gonochorists, and either or both can coexist with various types of hermaphrodites in a mixed sexual system (Fig. 4C). For instance, caridean shrimp have six sexual systems, including gonochorism (Fig. 4C6), protandry (Fig. 4C4), protandric simultaneous hermaphroditism (Fig. 4C5), and mixtures of protandry with males or females (Fig. 4C7 and 8) (Baeza 2018). Pedunculate barnacles have three sexual systems, including outcrossing simultaneous hermaphrodites (Fig. 4C2), simultaneous hermaphrodites with dwarf males (androdioecy, Fig. 4C9), and females with dwarf males (Yusa et al. 2012). Other androdioecious species, including the nematode *Caenorhabditis elegans* and an invasive insect, the cottony cushion scale, have self-fertilizing hermaphrodites and males (Cutter et al. 2019; Mongue et al. 2021). Gynodioecy, in which females coexist with hermaphrodites (Fig. 4C10), is widespread and common in flowering plants (Jacobs and Wade 2003). Populations of the mussel *Semimytilus algosus* include simultaneous hermaphrodites, males and females (trioecy, Fig. 4C12) (Oyarzun et al. 2020). Changes in sex allocation with growth, development, and/or social context can generate mixtures of males or females and hermaphrodites. For instance, at high density, seabass *Serranus psittacinus* groups include phenotypic hermaphrodites and males. Small hermaphrodites function as females; large hermaphrodites spawn as males with small ones and as females with the largest individuals, which have become fully male (Fig. 4C13) (Petersen 2006). Moreover, multiple types of hermaphrodites can coexist. In *Lythrypnus* gobies, along with a capacity for bidirection sex change, several species have mixed sex allocation patterns (St. Mary 2000); these include combinations of pure males and females with female-biased hermaphrodites (Fig. 4C14), of male- and female-biased hermaphrodites with pure females (Fig. 4C15), and of male- and female-biased hermaphrodites (Fig. 4C11). This nonexhaustive sampling of sexual systems illustrates the diversity in organization of sex3 considered from the perspective of unitary organisms, such as humans or fruit flies, that have developmentally, structurally, and functionally coherent individuals (Vuorisalo and Tuomi 1986).

However, for modular, clonal, or colonial organisms—such as plants or corals—with independent or semi-independent subunits, we need to consider sex3 across subunits at multiple levels of organization, adding substantial spatiotemporal complexity to sexual systems (Chen and Pannell 2025a, Chen and Pannell 2025b; Casper 2026; Casper and Warkentin 2026). While taxon-specific vocabulary exists for parts of this complexity (Jackson 1928; Wasson and Newberry 1997; dos Santos et al. 2026), there is no general framework for multilevel sex3 across eukaryotes. Moreover, in life cycles with alternation of generations, body organization and sexual systems may differ between generations. For instance the polyps of a simultaneously hermaphroditic hydrozoan colony may give rise to gonochoristic male and female medusae (Siebert and Juliano 2017). Sex3 may also be determined and expressed in different generations. For instance, diploid sporophytes of seed plants meiotically produce micro and/or megaspores, and their haploid gametophyte progeny produce micro- and/or macrogametes (Petersen and Burd 2017). Both generations use sexual reproductive processes, and may be sexually differentiated, but standard diplontic terminology is inadequate to describe them; three- and four-sex systems have been suggested (Meissner 2021). In summary, as unitary diplontic gonochorists, humans occupy the smallest, simplest corner of an expansive sex3 conceptual universe. Research on organisms with greater complexity and diversity in sex3 is enriching our understanding of the realm of possibility, and yielding insights into both patterns and mechanisms of evolution and development of sex3.

### Sex4. Traits associated with anisogamete type/s produced

Sex4 includes species-specific sets of genetic and phenotypic characteristics associated with anisogamete production strategy. Sex4 traits can be associated with sex3 for multiple developmental, functional, and evolutionary reasons. For instance, they may be involved in sex3 determination. Diverse genetic mechanisms regulate sex3 expression in many gonochoristic and mixed sexual systems (Bachtrog et al. 2014; Fusco and Minelli 2019). In contrast, size and social dominance regulate sex change in many sequential hermaphrodites (Casas and Saborido-Rey 2021). Sex4 traits can be directly involved in producing anisogametes (e.g., differentiated gonads or gametangia) or in releasing, transferring, receiving, and retaining gametes (e.g., genitalia and sperm-storage organs). Sex4 traits may also function in postzygotic parental investment, such as egg care, gestation, or lactation in animals, the provisioning of moss sporophytes by gametophytes, and fruiting or other mechanisms of seed dispersal in flowering plants. Many sex4 traits have roles in signaling to potential mates and/or rivals, as do bird songs and plumage ornaments, or in receiving such signals, as do moth antennae (Rosenthal 2017). Others, such as the floral displays of plants, signal to gamete-transfer intermediaries. Sex4 traits need not be directly related to reproduction; for instance, some species have sex3-differentiated dietary, habitat, or lifestyle specializations. Moreover, some sex4 traits are involved in the development, maturation, and function of other sex4 traits; these include sex3-differentiated hormonal and gene expression profiles.

Many sex4 traits are commonly used to assess or assign sex, with the specific trait/s used varying with species and context; an animal might be sexed by size, color, or behavior in the field, genital inspection in the hand, or genetic markers in a tissue sample. These traits are correlated with sex3 gamete production, and with each other, but not perfectly. For instance, many birds are sexually dichromatic, so their coloration is a sex4 trait. However, plumage can vary developmentally, seasonally, and with individual condition. In addition, some species have sex-limited polymorphisms (Mank 2023; Falk 2026). For instance, in about 40% of dichromatic hummingbird species at least 10% of females express male plumage characteristics (Diamant et al. 2021). There can also be continuous variation of sex4 traits within or between morphs and sexes. For instance, in orangutans, some adult males express enlarged cheek pads (flanges) and are twice the size of adult females, while unflanged males are often similar in size to females; however, some unflanged males grow larger and may become flanged, and flanges can diminish in older males (Kralick et al. 2023). The cross-sexual transfer of trait expression can generate sex-limited polymorphisms, sexual monomorphisms, and reversals in trait expression between sex3 types (West-Eberhard 2003; Anderson and Falk 2023). For instance, parental care traits exhibit great diversity and evolutionary lability in their associations with sex3 among species, as exemplified by the many gains and losses of care, variation in care type, and transitions among male, female, and biparental care in amphibians (Furness and Capellini 2019).

The associations of sex4 traits with sex3 and each other vary. For sets of sex4 traits closely linked to sex determination and reproduction (e.g., genes, gonads, and genitals; Joel 2012), common combinations are called endosex and rare ones intersex. There is great diversity across intersex and endosex development (Ainsworth 2015), and this variation can enable evolutionary change. For instance, most placental mammals have chromosomal sex determination with XY males and XX females. However, functional phenotypic females with XY genotypes have evolved six times in *Akodon* mice, with an incidence of 10–66% of females across eight species (Hoekstra and Edwards 2000). In the African pygmy mouse, over 70% of females are XY and maternal care strategies differ by genotype, with XX females being more likely to engage in biparental care and XY females caring alone (Veyrunes et al. 2010; Heitzmann et al. 2023). Even in humans, some XY females can reproduce (Dumic et al. 2008). Discordance of sex4 genotype with sex3 gamete production and sex4 phenotypic traits has been documented in many vertebrates, and the effects on fitness vary (Wild et al. 2022; Bókony et al. 2025; Hall et al. 2025). Moreover, traits considered intersex in many species can be sex-typical in related species. For instance, in several species of moles all functionally female individuals have ovotestes (Carmona et al. 2008) and it seems that all red-tailed boa constrictors have vaginal pouches, not just individuals with ovaries (Brennan 2026, personal communication).

Overall, suites of sex4 traits, their distributions, and the strength and structure of their association with sex3 vary among lineages—across origins of anisogamy, through sex3 diversification, and among species. Unlike sex2, sex4 is not binary. Even in gonochoristic species, sex4 traits may be bi- or multimodal and individual development generates unique trait mosaics, or positions in the multivariate sex4 hyperspace (Hyde et al. 2019; McLaughlin et al. 2023). While sex4 is conceptually anchored in the associations of nongametic traits with gamete type/s, its composition is highly variable and its edges are fuzzy. The concept of sex4 recognizes the importance of suites of nongametic, sex-associated traits—including traits commonly used to assess sex—as well as the fact that these are not interchangeable with or equivalent to sex2 or 3.

### Sex states 2–4: Gamete traits, gamete-production strategies, and associated traits

Because many sex4 traits are more evident than gametes, humans and other organisms often use them to assess and categorize sex. Such categorization is a process that happens within the assessor, influenced by context and perception; it is not a property of assessed individuals (Rosenthal 2017), although it does evolve in response to sex4 changes in the assessed that alter correlations with gamete type (Lerch and Servedio 2023; Powell and Uy 2023). Nonetheless, a social and scientific emphasis on categorization fosters both essentialist reasoning (Saguy et al. 2021) and the idea that sex is fundamentally a category, not a complex, interacting network of biological states and processes that develop and evolve. Scientific debate on the non/binary nature of sex (reviewed in Ritz and DuBois 2025) largely reflects a tension between anisogamete-based definitions (sex3; Goymann et al. 2023; Griffiths and Spencer 2025) and multivariate models that include sex3 but are dominated by sex4 (Massa et al. 2023; McLaughlin et al. 2023). In practice, attempts to define sex solely through a binary gametic focus are easily oversimplified, often mixing trait-based convergent evolution with lineage-based continuity of sex2 and minimizing the complexity that sex3 introduces across lineages. Focusing on a multivariate sex hyperspace that includes gamete size makes sense developmentally and phenotypically, but not for evolutionary analyses of the origins or consequences of anisogamy. Moreover, framing gamete type as part of a variable constellation of sex-associated traits suggests sex operationalization is arbitrary. This may be true in a human, social context (Massa et al. 2023; Velocci 2024; Watkins and DiMarco 2025), and other organisms also assess sex in diverse ways, but the evolution of sex-assessment strategies is fundamentally shaped and constrained by gamete compatibility.

Collapsing sex2–4 into a single concept that can be framed or operationalized in different ways is insufficient—although this is how people often use *sex*. It leads to unnecessary argument about the “correct” meaning of *sex/es*. It fosters perceptions that sexes are arbitrary human constructions, or simply systems of categorization. It also suggests that information can be generalized across the entirety of sex2–4, hiding real biological complexity. We have found that conceptually separating these three sex-state concepts is useful to acknowledge their distinct biological properties and complexities, while also clarifying and facilitating discussion of their interrelatedness.

## Actions and interactions of gamete producers

### Sex5. Syngamy-facilitating actions

Sex5 includes the actions and interactions of gamete-producing organisms that enable encounters between compatible gametes. It is a key prerequisite for syngamy (part of sex1) to occur because gametes themselves have limited lifespans, mobility, and environmental tolerance and—unlike some forms of sex0—syngamy requires cell contact. Animal copulation is one form of sex5, often called *sex* in recognition of its key reproductive role, but there are an array of more specialized terms for the ways other eukaryotes achieve gamete encounters (Fusco and Minelli 2019). We include sex5 in our framework to encourage broader comparisons of this crucial concept in the biology of sex, beyond taxonomic silos. Sex5 actions may be associated with sex3 and 4, but need not be; isogamete producers also engage in sex5.

In unicellular eukaryotes, syngamy is infrequent and often impossible. To enable it, these organisms must either develop into or produce syngamy-capable cells, which occurs only under specific conditions (Rizos et al. 2024; Constable and Liu 2026). Their transition to a sexual phase and preparation for syngamy can be coordinated through a shared response to stressors or cues (Levin and King 2013; Woznica et al. 2017), and in some species occurs only after individuals aggregate or pair (Rizos et al. 2024). Such actions are as necessary for syngamy as the transfer of gametes between reproductively compatible animals through copulation.

Multicellular eukaryotes have diverse bodies, lifestyles, and strategies for facilitating gamete encounters. Animals often transfer gametes directly into or onto a partner’s body. They also deposit and pick up spermatophores with varying degrees of coordination, coordinate gamete release in broadcast and pair or group spawning, and use spermcasting and filtration (Fusco and Minelli 2019). Plants also use a variety of mechanisms to bring gametophytes and gametes into proximity. Angiosperms have repeatedly evolved mechanisms to attract, reward, and manipulate pollinators (Barberis et al. 2023; Liao et al. 2025) and have diverse and complex gametic and gametophytic communication systems (Zheng et al. 2024; Becker et al. 2025). Gymnosperms make specialized fluids to capture pollen, transport it to the ovule, regulate its germination, nourish male gametophytes, and reward pollinating insects (von Aderkas et al. 2018; Prior et al. 2019). In other land plants with free-swimming sperm, the archegonia that make eggs also release chemicals to attract sperm (Becker et al. 2025). Bryophytes and red algae use arthropods, as well as water, to transport their sperm (Rosenstiel et al. 2012; Lavaut et al. 2022; Deregnier et al. 2025). Moeover, fungi have also evolved multiple ways to enlist animals to transfer their gametes, including manipulating plants to grow pseudoflowers that present fungal spermatia rather than pollen (Bultman et al. 1998; Cano et al. 2013; Slot and Kasson 2021). Across eukaryotes, there is a rich evolutionary history and amazing diversity of solutions to the challenge of facilitating gamete encounters.

### Sex6. Nonreproductive expressions and derivatives of syngamy-facilitating actions

Sex6 includes byproduct expression of syngamy-facilitating actions in nonreproductive contexts as well as evolutionary derivatives of such traits that may have additional or different functions. Traits that evolved under selection for a specific function, in a particular context, can be expressed in other contexts for many reasons. Some of these are incidental byproducts, and they may persist as such if they are selectively neutral or constraints prevent more targeted expression. However, traits often gain new functions, and selection in new contexts leads to changes in their form and regulation (West-Eberhard 2003). Such coopted or repurposed traits are called exaptations (Gould and Vrba 1982). Evolution is shaped by historical contingency, and traits can change or add functions many times (Prum 2005; Terrill and Shultz 2023). Moreover, novelties are often built from pre-existing parts (Linz et al. 2020). Sex6 recognizes the role of sexual traits in such evolutionary dynamics.

Most of the human behavior that we call *sex* is nonreproductive, and there is a deep primate history of multifunctional, multicontextual use of behavior identical to, or derived from, syngamy-facilitating actions (Dixson 2012; Coxshall et al. 2026). Across animals, sex5 behavior is expressed in many nonreproductive contexts, including with different-sex, same-sex, heterospecific, and deceased partners, in groups, and alone (Gunst et al. 2018; Pintanel et al. 2021; Würsig and Orbach 2023; Villar 2024; Soni et al. 2025). For instance, most mammals only copulate during a short estrus period near ovulation, when conception is possible (sex5). However, some have concealed ovulation and extended periods of sexual activity, so that most of their copulations are nonreproductive (sex6). This enables females to mate with many males, confusing paternity, which can improve infant survival by decreasing infanticide by males—a major source of infant mortality in some species (Wolff and Macdonald 2004; Fürtbauer et al. 2011; Lukas and Huchard 2014). Same-sex sexual behavior (SSB) is documented in over 1500 animal species (Monk et al. 2019; Gómez et al. 2023). In some cases, this may be a byproduct of adaptive mating strategies, if sex discrimination is difficult or costly or if the risk of missed opportunities exceeds the cost of SSB (Lerch and Servedio 2023; Lerch 2026). For instance, male squid deposit spermatophores indiscriminately on males and females (Hoving et al. 2012; Hoving et al. 2019). In primates, SSB is correlated with promiscuity and may be a neutral byproduct of selection for greater mating activity (MacFarlane and Vasey 2016) or simply pleasure-seeking (Cunningham and Benítez 2024). However SSB—and sex6 more broadly—also mediates cooperation and affiliation in prosocial species, including chimpanzees and bonobos (Moscovice et al. 2019; Sandel and Reddy 2021; Brooker et al. 2025). SSB facilitates same-sex pair-bonding and coparenting in many birds, allowing successful reproduction in sex3-skewed populations (Young and VanderWerf 2014; Gillies and Siddiqi-Davies 2025). Sexual behavior has even been coopted for thermoregulation and defense. Red-sided garter snakes emerging from hibernation underground are cold and vulnerable to predation by crows; some cold males facultatively express female pheromones, attracting courtship from other males that warms and protects them (Shine et al. 2012). While research on sex6 is growing, the full amount of nonreproductive sexual behavior in animals is still unknown. For instance female-on-male mounting in birds—without cloacal contact that would enable sperm transfer—appears to be widespread and accounts for as much as 25% of mounting in some species; it has received little attention and is likely underreported although, like SSB, it is probably adaptive in some contexts (Villar 2024). The scientific history of viewing nonreproductive expressions and derivatives of syngamy-facilitating actions, particularly in animals, as necessarily deleterious has limited our understanding of them but not their evolution.

In plants, many traits that play a direct role in facilitating gamete encounters also have other reproductive and nonreproductive functions. In some cases, initially nonreproductive traits have gained roles in syngamy-facilitation. For instance, secondary metabolites in nectar that improve pollinator performance may have evolved as antiherbivore defenses (Barberis et al. 2023) and extrafloral nectaries have adapted for pollinator attraction and reward (Knox et al. 1985). In other cases, syngamy-facilitating traits have gained or changed function. For instance, the common ancestor of *Dalechampia* vines evolved resin-secreting flowers that reward resin-collecting bee pollinators, possibly by coopting defensive resins of a more distant ancestor, and resins have been subsequently redeployed for defense multiple times, in a variety of tissues (Armbruster et al. 2009). In some orchids, flowers have evolved to mimic wasps or bees, visually and chemically attracting males of those insects to pollinate them. These traits function in gamete-transfer (sex5) and may also function to repel herbivores, as a form of Batesian mimicry (Lev-Yadun and Ne’eman 2012). Similarly, the carrion and dung odors of some flowers that attract flies and beetles as pollinators may also repel mammalian herbivores (Lev-Yadun 2019). Traits can change function multiple times, gaining and losing syngamy-facilitating and other roles. For instance, floral bracts serve multiple functions in >80% of species examined (Song et al. 2024). They are known for their importance in signaling to pollinators. However, they also protect against flower, fruit and seed herbivores, shelter gametophytes and/or pollinators from abiotic stressors, photosynthetically supply resources for postzygotic development, and aid in seed dispersal (Song et al. 2024). Plant traits that gain or originate for syngamy-facilitating roles are not thereby constrained from adding or changing functions as evolution continues.

Like other traits, Sex5 traits can exapt in a range of reproductive and nonreproductive ways. Assuming otherwise has limited research paradigms and biological understanding, such as framing SSB as inherently paradoxical (Monk et al. 2019; Lerch 2026). Many traits are clearly multifunctional, like limbs or sensory systems, and are used in reproductive and nonreproductive behaviors and life stages. Traits that evolved for the purpose of reproduction can also have nonreproductive functions. For instance, in many wasps, ovipositors serve the dual functions of egg-laying and venom injection, both for defense and to manipulate hosts during parasitization (Özbek et al. 2019). In many plants, reproductive organs such as flowers, fruits, and seeds can photosynthesize and may contribute substantially to the plant’s carbon gain (Brazel and O’Maoiléidigh 2019). Multifunctionality may play an important role in organismal interactions and provide a source for evolutionary novelty. For example, cerci—paired appendages on the rear of many arthropods—are often used in courtship as sensory organs or for sperm transfer. In earwigs, cerci are still used by both sexes during copulation (Briceño and Eberhard 1995) but have evolved to become forcep-like and gained functions in intrasexual competition and antipredator defense (Eisner 1960). The relationship between sex5 and sex6 fits within the larger framework of trait multifunctionality.

## Conclusions: The depth and breadth of sex through the lens of multiple sex concepts

From the ancient, diverse genetic recombination of sex0, there was a single origin of sex1. Meiosis required syngamy restriction and syngamy needed facilitation, which led to multilayered evolutionary diversification. Syngamy restriction led to lineage divergence, mating types, and many convergent origins of sex2 with similarities, differences, and varying levels of stability, elaboration, and loss. The organization of sex2 expression diversified, generating many sex3 strategies and evolutionary flux among them. Sex3 generated lineage-specific multivariate hyperspaces of sex4 traits, with varied distributions and associations. Organisms evolved diverse sex5 actions and interactions to facilitate syngamy, with unique and convergent features. Some sex5 traits were expressed in nonreproductive sex6 contexts, sometimes gaining new functions. The depth and breadth of this evolutionary history is reflected in a great diversity of manifestations in extant life forms. We offer these sex concepts as an open framework to acknowledge the scope and discuss the complexity of sex in biology.

The challenge of understanding the history and diversity of sex has motivated a huge body of biological research. By necessity, much of this is deeply specialized, focused on specific questions and particular taxa. Only small fractions of this research reach a broader range of biologists, our students, and the general public, through filtering highly shaped by social factors and human interests (Donovan et al. 2024). This has fostered oversimplification and overgeneralization about sex that does not reflect the richness and complexity of biology, the dynamic progress of science, or the collective understanding of those who study sex. Unfortunately, this narrowed view of biology is currently being wielded to justify social harms (Ritz and DuBois 2025). While focus and filtering are necessary for synthesis, integration, pedagogy, and more, how we frame the results matters. Situating focal subsets, teachable simplifications, or examples within a more expansive conceptual framework can limit overgeneralization, fostering curiosity and broader thinking (Dauer and Dauer 2016).

Viewing sex concepts as anchor points, not bounded definitions, can facilitate consideration of the biologically fuzzy edges generated through evolutionary changes and individual developmental variation. This variation offers excellent research opportunities but requires careful dissection and accommodates multiple operationalizations of sex. We discussed this for sex1, and it has been addressed extensively for sex4 (Velocci 2024; Ritz and DuBois 2025; Watkins and DiMarco 2025). Mating types and isogamous descendants of anisogamous lineages could be framed as fuzzy edges of sex2. The framework of phenotypic and functional quantitative gender accommodates continuous variation within sex3, while the diversity of sex3 expression patterns means any categorical structure will have fuzzy edges. Sex5 involves fuzzy overlap between traits of gametes and gamete-producers, as well as with sex6. Anchor points offer structure for considering relationships and exploring variation, without requiring biologically unrealistic boundaries or dividing organisms into those that fit within our concept and others that do not.

Our consideration of sex concepts highlights some of the challenges humans face in attempting to understand biology across the breadth of organismal diversity. We begin from a lay understanding based on our own biology and inflected through the lens of our social history, as did others before us upon whose work we build. We study organisms like and unlike ourselves, asking questions shaped by those starting points and our observations. We apply words used loosely in everyday life within scientific contexts that demand greater clarity, which seems to work for some organisms but nonetheless shapes our thinking about them. The kind of meaning slippage that may delight in literature confounds trait-based analogy and lineage-based homology of sex2, obscuring variation in multiple ways. Similarly, the conceptual collapse of sex2–4 generates confusion and fosters oversimplification. Deeper knowledge, especially of organisms unlike us, necessitates invention of new language, which often remains largely restricted to specialized communities, although more common language fails to accurately represent those organisms. We discussed examples in sex1–3. The silos generated by increasingly specialized knowledge limit broader comparisons; for instance, much of what we include in sex5 is rarely addressed in an integrated manner. This is unfortunate as understanding organisms unlike ourselves is particularly useful both for perspective on our own biology and to develop a more expansive view of life. Moreover, as unitary gonochorists who only reproduce sexually, living in societies that emphasize, structure and regulate sex3–6, we often view sex as special, failing to apply our broader understandings of evolution and development to sexual traits (Ah-King and Nylin 2010; Anderson and Falk 2023; Falk 2026; Lerch 2026).

In biology, *sex* means many things, reflecting the importance, deep history, and diverse manifestations of a suite of related phenomena. Socially, sex is a topic of intense interest and contested meaning, where “the biological” has particular power. Both socially and to support the growth of scientific understanding, biologists have a responsibility to represent our knowledge about sex in clear and accessible ways without misleading oversimplification. Attempts to claim a single concept as the only true “biological sex” both diminish the scope of sex in biology and support social harm, even if unintentionally. In the 20th century, the “species problem” in biology spawned many competing species concepts and arguments over which was best. In the 21st century, this has been largely resolved with the recognition that both our theoretical understanding of species and the operational criteria most useful to recognize and study them are heterogenous, and this is not a problem but rather a reflection of the complexity and dynamic nature of their biology (Hey 2006; Haveman 2013; Sandberg et al. 2025). Sex encompasses a greater breadth of biological phenomena than species; it is multifaceted, heterogenous, and complex and this is not a problem. We do, however, need ways to acknowledge the breadth and discuss the complexity of sex more clearly. Our framework for multiple sex concepts recognizes the many distinct ways we use *sex*, makes space for fuzzy edges, and highlights challenges, offering a scaffold to facilitate broader thinking, better communication, and discovery.

## Notes

From the symposium “Sex Across Origins: Questioning Animal-centric Assumptions and Developing Integrative Frameworks” presented at the annual meeting of the Society for Integrative and Comparative Biology, January 3-7th, 2026.
